# Optimization of agro-residues as substrates for *Pleurotus pulmonarius* production

**DOI:** 10.1186/s13568-019-0907-1

**Published:** 2019-11-14

**Authors:** Nan Wu, Fenghua Tian, Odeshnee Moodley, Bing Song, Chuanwen Jia, Jianqiang Ye, Ruina Lv, Zhi Qin, Changtian Li

**Affiliations:** 1 Engineering Research Center of Edible and Medicinal Fungi, Ministry of Education, Jilin Agricultural University, Changchun, 130118 China; 2 Institute of Microbiology Guangxi Academy of Agricultural Sciences, Nanning, 530007 Guangxi China; 3 College of Life Sciences, Jilin Agricultural University, Changchun, 130118 China

**Keywords:** *Pleurotus pulmonarius*, Mushroom crop, “Replacing wood by grass”, Simplex-lattice design, Cultivation, Biological efficiency

## Abstract

The “replacing wood by grass” project can partially resolve the conflict between mushroom production and balancing the ecosystem, while promoting agricultural economic sustainability. *Pleurotus pulmonarius* is an economically important edible and medicinal mushroom, which is traditionally produced using a substrate consisting of sawdust and cottonseed hulls, supplemented with wheat bran. A simplex lattice design was applied to systemically optimize the cultivation of *P. pulmonarius* using agro-residues as the main substrate to replace sawdust and cottonseed hulls. The effects of differing amounts of wheat straw, corn straw, and soybean straw on the variables of yield, mycelial growth rate, stipe length, pileus length, pileus width, and time to harvest were demonstrated. Results indicated that a mix of wheat straw, corn straw, and soybean straw may have significantly positive effects on each of these variables. The high yield comprehensive formula was then optimized to include 40.4% wheat straw, 20.3% corn straw, 18.3% soybean straw, combined with 20.0% wheat bran, and 1.0% light CaCO_3_ (C/N = 42.50). The biological efficiency was 15.2% greater than that of the control. Most encouraging was the indication that the high yield comprehensive formula may shorten the time to reach the reproductive stage by 6 days, compared with the control. Based on the results of this study, agro-residues may be used as a suitable substitution for sawdust and cottonseed hulls as the main cultivation substrates of *P. pulmonarius*. These results provide a theoretical basis for the “replacing wood by grass” project on edible mushroom cultivation.

## Introduction

This study describes a comprehensive program to utilize herbaceous plants (mainly grasses) to grow edible and medicinal mushrooms, and produce fungi forage and fungi fertilizers with agricultural residues as substrates. “Replacing wood by grass” has many apparent advantages, such as the high utilization of solar energy and resources, lower costs, and shorter production periods to name a few (Sanderson and Reed 2000; Springer et al. 2003). Applying the “replacing wood by grass” system to develop a more ecologically conscious mushroom industry may not only resolve the conflict between mushroom production and ecosystem balance, but it may also lead to great improvements in soil and water conservation, ecological environmental protection, increases of food resources, and the promotion of a sustainable agricultural economy (Lin 2009).

*Pleurotus pulmonarius* (Fr.) Quél., also known as anchovies and Indian abalone mushrooms (Li et al. 2015), is most commonly known as the grey oyster mushroom, which is characterized by a grayish colored sporophore, which has a fleshy texture and produces an aromatic, not anise-like aroma (Lechner et al. 2004). *P. pulmonarius* is formally classified in the following categories: Fungi, Basidiomycota, Agaricomycetes, Agaricomycetidae, Agaricales, Pleurotaceae, Pleurotus (http://www.indexfungorum.org/Names/Names.asp). *P. pulmonarius* has an outstanding reputation as an edible mushroom that has been used for centuries as a food and food flavoring because of its highly desirable taste qualities and unique aroma (Oliveira et al. 2002; Zhang et al. 2013). It also contains a large range of proteins, polysaccharides, essential amino acids, a high potassium to sodium ratio, and multivitamins, including niacin and riboflavin (Velázquez-Cedeño et al. 2002; Stanley et al. 2011). *P. pulmonarius* is widely marketable and sells well in southeast Asia, Japan, and several other countries. However, it is more expensive than other mushrooms, such as *Flammulina velutipes* and *P. ostreatus*. Recently, many studies have confirmed that *P. pulmonarius* has pharmacological properties, such as antioxidants, anti-cholinesterase, antitumor, antibacterial, immuno-modulating, anti-inflammatory, aids in resisting potential vascular complications, and may reduce blood sugar levels (Olufemi et al. 2012; Smiderle et al. 2012; Xu et al. 2012; Wahab et al. 2014; Ge 2015; Nguyen et al. 2016; Ni 2016; Zhang et al. 2016).

Sawdust and cottonseed hulls are the main materials for the traditional cultivation of *P. pulmonarius*. However, the price of cottonseed shells has risen to 282 US dollars per ton (Liu et al. 2011). Complicating the matter, the logging of trees has been declared as a social, environmental, and global issue. In China, there are abundant agro-residue resources, such as wheat straw, corn cobs, rice straw, soybean straw, rice straw, peanut straw, rape straw, and corn straw. Substrates play an indispensable role in the nutritional composition of oyster mushroom and previous studies have found astounding differences in both proximate and mineral composition of *Pleurotus* when these mushrooms are raised in different substrates (Sarker et al. 2007; Bhattacharjya et al. 2015). The great potential of this industry is derived from its promise of saving land and labor, its eco-friendly nature, and its low required startup capital. For these reasons, it is urgent to find new substrates for mushroom crop cultivation in relation to the “replacing wood by grass” project.

The objectives of this study were to evaluate different agro-residues as potential substrates for *P. pulmonarius* cultivation using a simplex-lattice design method, optimize substrate composition, and evaluate the combined effects of the substrate on mycelial growth rates, yield, and fruiting body traits of *P. pulmonarius*. This study was also intended to provide information for the further development of the “replacing wood by grass” project in China by investigating a variety of agro-residues that could replace sawdust and cottonseed hulls in *P. pulmonarius* cultivation.

## Materials and methods

### Microorganism and spawn preparation

Mycelia of *P. pulmonarius* CCMJAU 201708 were obtained from the Engineering Research Center of the Chinese Ministry of Education for Edible and Medicinal Fungi of Jilin Agricultural University, China. Mycelia were cultured on potato dextrose agar (PDA) in a Petri dish for 6 days at 24 °C in darkness. When mycelia had grown to completely cover the media, mycelia with a diameter of 5 mm were collected for use as future inoculum sources.

Liquid spawn was prepared by culturing mycelial plugs (2 mm in diameter, 10 plugs/flask) in 1 L flasks containing 400 mL of liquid media, while shaking (150 rpm/min) in a rotatory incubator at 24 °C for 9 days, in darkness. Liquid media consisted of maize meal (40 g/L), glucose (20 g/L), peptone (1 g/L), KH_2_PO_4_ (10 g/L), and MgSO_4_ (5 g/L) at a of pH 6.5 (Wu 2011).

### Substrate preparation

Various agro-residues, including wheat straw, rice straw, soybean straw, corn straw, corn cobs, peanut straw, rape straw, and supplements, including wheat bran and light CaCO_3_, for the cultivation of *P. pulmonarius* were purchased from the mushroom base of Jilin Agricultural University, Changchun, China. All agro-residues were chopped, air dried, and ground into a fine powder.

### Screening of appropriate agro-residue substrates in Petri dishes

The CK (control) substrate, or standard formulation, for screening and comparing additional substrates was comprised of 40.0% sawdust, 39.0% cottonseed hulls, 20.0% wheat bran, and 1.0% calcium carbonate (Fan et al. 2016). In the additional substrate formulations, or the treatment groups, one of seven kinds of agro-residues (wheat straw, rice straw, soybean straw, corn straw, corn cobs, peanut straw, rape straw) was used to make up 79.0% of the total substrate by replacing sawdust and cottonseed hulls. All the substrates were pressed into separate Petri dishes (90 mm in diameter) to a height of 5 mm and autoclaved at 121 °C for 1 h.

Mycelial plugs, 5 mm in diameter, were cut from the leading edge of actively growing cultures on PDA plates. One plug was inoculated into the center of each of the Petri dishes containing the various agro-residue substrates. The Petri dishes were incubated in darkness at 24 °C for 7 days. Each treatment was replicated five times. Mycelial growth rates were measured as subsequently described. Every day, the radial growth of each treatment was observed. The diameter of each colony was measured on two perpendicular axes bisecting the center of the colony, then the mean value over time was calculated to reflect the growth rate of the mycelia. The three agro-residues associated with the fastest mycelial growth rates were then considered in further analyses.

### Substrate formulations

A one-factor-at-a-time (OFAT) method (Singh et al. 2011) was used to determine the initial range of the three independent variables. Based on results from the Petri dishes, the variables X_1_ = wheat straw, X_2_ = corn straw, and X_3_ = soybean straw were selected for a simplex-lattice design. These were then mixed in various combinations to determine the optimal substrate composition for the cultivation of *P. pulmonarius*.

$$0\% \, \le \,{\text{X}}_{ 1} \, \le \, 100\% ,{\text{ wheat}}\;{\text{straw}}$$

$$0\% \, \le \,{\text{X}}_{ 2} \, \le \, 100\% ,{\text{ corn}}\;{\text{straw}}$$

$$0\% \, \le \,{\text{X}}_{ 3} \, \le \, 100\% ,{\text{ soybean}}\;{\text{straw}}$$

$${\text{X}}_{ 1} \, + \,{\text{X}}_{ 2} \, + \,{\text{X}}_{ 3} \, = \, 100\%$$

Ten possible mixture formulations were designed with the main ingredient accounting for 79.0% of the total substrate (Table 1). The substituting ingredients were wheat straw, corn straw, and soybean straw, as previously described. The main ingredient of the control substrate mixture (CK) was composed of 40.0% sawdust and 39.0% cottonseed hulls. The remainder of all formulations consisted of 20.0% wheat bran, 1.0% light CaCO_3_, and water at a pH of 7.0. Environmental conditions were kept constant across all treatments and each treatment was replicated three times.

**Table 1:** Experimental design and model formulations for cultivation materials

Formulation	Substrate mixture ratio		
	X_1_ (%)	X_2_ (%)	X_3_ (%)
1	100	0	0
2	0	100	0
3	0	0	100
4	50	50	0
5	50	0	50
6	0	50	50
7	33.3	33.3	33.3
8	16.7	16.7	66.7
9	16.7	66.7	16.7
10	66.7	16.7	16.7

X_1_-wheat straw; X_2_-corn straw; X_3_-soybean straw

### Model analysis

The Scheffé model was fitted using a polynomial quadratic equation in order to correlate the response variable (Y) with the independent variables (X). The Eq. was as follows:

$${\text{Y = }}\sum\limits_{1 \le i \le q} {\beta_{i} X{}_{i}} + \sum\limits_{1 \le i \le j \le q} {\beta_{i} X{}_{i}X{}_{j}}$$

The mixture experiment and optimization design followed previously described methods (Scheffe 1963; Lundstedt et al. 1998; Yang et al. 2016; Song et al. 2018) (Table 1).

### Cultivation method

Substrates were prepared from the various agro-residue components as 79.0% of the main substrate, supplements (wheat bran and light CaCO_3_) as 21.0% of the substrate mixed with sterilized water was added to ± 72.5%. A total of 500.0 g of each of the substrates (dry weight of 150.0 g) was used to fill a 20 cm × 17.5 cm × 0.05 cm polypropylene bag, with a 2.5 cm collar at the bag mouth. The filled bags were then autoclaved at 121 °C for 1 h. After cooling below 20 °C, each bag was then inoculated with 15 mL of liquid spawn of *P. pulmonarius*.

After inoculation, all bags were incubated in the dark at 22–24 °C, at a carbon dioxide (CO_2_) concentration below 2500 ppm. After it mycelia filled the bags, each formulation was cultivated for four additional days to ensure physiological maturity. Subsequently, each bag was cut to create two “V” shaped holes on the surface using a sterilized knife. The bags were then transferred to a cultivation room. All treatments were maintained in the same room under the same conditions (15–17 °C, 90–95% relative humidity, 400–700 ppm CO_2_ concentration, under a 200 LUX light source for 10 h/day) to facilitate basidiocarp growth. Mushroom harvest times were recorded as they occurred. After harvest, the mushroom yield corresponding to each bag was weighed. The harvest included 20 bags from each treatment with three replicates; all data were used for statistical analyses.

### Data collection

The radial growth of each formula in Petri dishes, time to bud formation, growth parameters pertaining to a single fruiting body (length and width of the stipe, and length, thickness, and width of the pileus measured using a Vernier caliper), time to harvest, and reproductive stage and yield parameters including total fresh weight (g) were collected. The biological efficiency (BE) for each substrate formulation was calculated as follows:

$$BE(\% ) = \frac{Fresh\;weight\;of\;mushroom}{Dry\;weight\;of\;substrate} \times 100$$

$$Reproductive\;stage\left( d \right) = Harvest\;time - Bud\;time$$

### Statistical analyses

Design expert statistical software package V 8.0.6.1 was used to construct a regression analysis. A quadratic multiple regression fitting of yield, mycelial growth rate, stipe length, pileus length, pileus width, and time to harvest against the substrate formula ingredients was performed using the Scheffé quadratic polynomial regression model. The regression equation for each evaluation index was established. The influence of the interaction between the three main ingredients on the evaluation index was analyzed. The optimization function was used to set the variation range and the expected response value for each ingredient. Beginning with a random combination, the steepest slope prediction was performed until the target response value was reached. The optimized yield formula derived from the regression analysis was verified, an analysis of variance (ANOVA) analysis was performed to estimate the statistical parameters using SPSS Statistics 17, and finally, the significant differences between the means were determined by a Student–Newman–Keuls test (Yang et al. 2016). Unless otherwise stated, the differences were considered statistically significant at *P* < 0.05.

### Verification test

The substrate formula, which was determined to be optimal according to the previous experiment, was tested against the CK substrate formula at the mushroom base at Jilin Agricultural University. The results were analyzed to determine the feasibility of using the treatment substrate formula in place of the CK.

## Results

### Screening results of agro-residue substrates

**Fig. 1:**
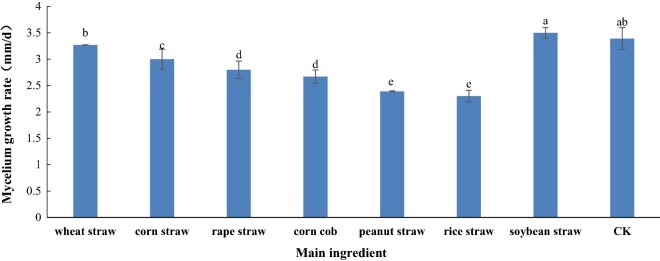
Effect of different agro-residue substrates on mycelial growth rate in Petri dishes. Mean values followed by no letters are not significantly different at a level of 5% (P < 0.05)

Based on the results obtained from screening the Petri dishes, there were significant (P < 0.05) differences between the various agro-residues (Fig. 1). Soybean straw was determined to be the best for the mycelial growth of *P. pulmonarius*. The mycelial growth rate in the soybean straw was significantly higher at 3.50 mm/day than in the CK, which was 3.39 mm/day. Wheat straw and corn straw followed, with mycelial growth rates of 3.27 mm/day and 3.00 mm/day, respectively. The mycelial growth rate in the rice straw was slowest at 2.30 mm/day. From these results, soybean straw, wheat straw, and corn straw were chosen as the appropriate agro-residue substrates for the subsequent D-optimum experiment.

### Effects of different agro-residue formulations on time to harvest

With the statistical analysis of the time to harvest for each substrate formula (Additional file 1: Table S1), the following regression model equation between the time to harvest and each ingredient was developed:

$$\begin{aligned} Y & = 42.74X_{1} + 42.41X_{2} + 36.08X_{3} + 4.97X_{1} X_{2} - 5.02X_{1} X_{3} + 11.64X_{2} X_{3} \\ & \quad - 29.33X_{1}^{2} X_{2} X_{3} - 304.75X_{1} X_{2}^{2} X_{3} - 17.31X_{1} X_{2} X_{3}^{2} \\ \end{aligned}$$

$$\left( {R^{2} = 0.8597} \right)$$

Using the regression model, the predicted time to harvest was calculated for each substrate formula. The linear correlation coefficient results for time to harvest was relatively high at R^2^ = 0.8597. The statistical analysis showed that the *P* values of both the mixed linear model and the quadratic regression model were < 0.0001, indicating that these two models were very significant and well fitted to the relationship between ingredients and time to harvest. Additionally, the correlation coefficient indicated that the equation model had an 85.97% goodness-of-fit to the experimental data. The analysis of variance (ANOVA) for *Y* was shown in Additional file 1: Table S1. The *P* value showed the probability of whether the time to harvest was affected by the different agro-residues. The terms which had *P* values < 0.05 had an important effect on the time to harvest. The coefficients of the independent variables in the equation reflect the degree of influence of the independent variable in the equation, i.e., the degree of contribution. As seen in the regression model, the equation coefficients were K (X_1_) = 42.74 > K (X_2_) = 42.41 > K (X_3_) = 36.08. Combined with the interaction analysis, and based on the f-value and *P* value, the interaction between X_2_X_3_ and X_1_X_2_^2^X_3_ significantly affected the time to harvest. It could be concluded that the time to harvest was changed by the appropriate selection of the X_1_, X_2_, and X_3_ levels. Interactions between X_1_X_2_^2^X_3_ may significantly reduce the time until harvest and interactions between X_2_X_3_ may significantly increase the time until harvest.

### Effects of different agro-residue substrate formulations on stipe length

With the statistical analysis of the stipe length for each substrate formula (Additional file 1: Table S2), the following regression model equation between the stipe length and each ingredient was developed:

$$\begin{aligned} Y & = 32.83X_{1} + 34.94X_{2} + 42.84X_{3} - 3.92X_{1} X_{2} + 23.33X_{1} X_{3} - 28.19X_{2} X_{3} \\ & \quad + 743.05X_{1}^{2} X_{2} X_{3} + 401.26X_{1} X_{2}^{2} X_{3} - 306.44X_{1} X_{2} X_{3}^{2} \\ \end{aligned}$$

$$\left( {R^{2} = 0.9241} \right)$$

The linear correlation coefficient for stipe length was relatively high at R^2^ = 0.9241. Additionally, the correlation coefficient indicated that the equation model had a 92.41% goodness-of-fit with the experimental data. As seen in the regression model, the equation coefficients were K (X_3_) = 42.84 > K (X_2_) = 34.94 > K (X_1_) = 32.83. These results indicated that soybean straw had the greatest degree of contribution to the stipe length of *P. pulmonarius*.

Combined with the interaction analysis, and based on the f-value and *P* value, the interactions between X_1_X_3_, X_2_X_3_, X_1_^2^X_2_X_3_, X_1_X_2_^2^X_3_, and X_1_X_2_X_3_^2^, were shown to have significantly affected stipe length (Additional file 1: Table S2). Interactions between X_2_X_3_ and X_1_X_2_X_3_^2^ may significantly reduce stipe length. Interactions between X_1_X_3_, X_1_^2^X_2_X_3_, and X_1_X_2_^2^X_3_ may significantly increase stipe length.

### Effects of different agro-residue formulations on pileus length

With the statistical analysis of the pileus length for each substrate formula (Additional file 1: Table S3), the following regression model equation between the pileus length and each ingredient was developed:

$$\begin{aligned} Y & = 35.06X_{1} + 48.00X_{2} + 50.20X_{3} + 6.65X_{1} X_{2} - 0.021X_{1} X_{3} - 17.22X_{2} X_{3} \\ & \quad + 768.97X_{1}^{2} X_{2} X_{3} - 422.50X_{1} X_{2}^{2} X_{3} + 389.72X_{1} X_{2} X_{3}^{2} \\ \end{aligned}$$

$$\left( {R^{2} = 0.9581} \right)$$

The statistical analysis showed that the *P* values of both the mixed linear model and the quadratic regression model were < 0.0001, which indicated that these two models were very significant and well fitted to the relationship between ingredients and the pileus length. Additionally, the correlation coefficient indicated that the equation model had a 95.81% goodness-of-fit with the experimental data. As seen in the regression model, the equation coefficients were K (X_3_) = 50.20 > K (X_2_) = 48.00 > K (X_1_) = 35.06. This result indicated that soybean straw had the greatest degree of contribution to the pileus length of *P. pulmonarius*.

Combined with the interaction analysis, based on the f-value and *P* value, the interactions between X_1_X_2_, X_2_X_3_, X_1_^2^X_2_X_3_, X_1_X_2_^2^X_3_, and X_1_X_2_X_3_^2^ were shown to have significantly affected the pileus length (Additional file 1: Table S3). Interactions between X_2_X_3_ and X_1_X_2_^2^X_3_ may significantly reduce the pileus length, and the interactions between X_1_X_2_, X_1_^2^X_2_X_3_ and X_1_X_2_X_3_^2^ may significantly increase the pileus length.

### Effects of different agro-residue substrate formulations on pileus width

With the statistical analysis of the pileus width for each substrate formula (Additional file 1: Table S4), the following regression model between the pileus width and each ingredient was developed:

$$\begin{aligned} Y & = 41.10X_{1} + 46.77X_{2} + 53.17X_{3} + 10.40X_{1} X_{2} + 4.52X_{1} X_{3} - 40.41X_{2} X_{3} \\ & + 889.89X_{1}^{2} X_{2} X_{3} - 573.34X_{1} X_{2}^{2} X_{3} + 86.31X_{1} X_{2} X_{3}^{2} \\ \end{aligned}$$

$$\left( {R^{2} = 0.9663} \right)$$

The ANOVA for *Y* is shown in Additional file 1: Table S4. The linear correlation coefficient for the pileus width was relatively high at R^2^ = 0.9241. As seen in the regression model, the equation coefficients were K (X_3_) = 53.17 > K (X_2_) = 46.77 > K (X_1_) = 41.10, which indicated that the degree of contribution of each ingredient to the pileus width was as follows: X_3_ (soybean straw) > X_2_ (corn straw) > X_1_ (wheat straw). This result indicated that soybean straw had the greatest degree of contribution to the pileus width of *P. pulmonarius*.

Combined with the interaction analysis, based on the f-value and *P* value, the interactions between X_1_X_2_, X_2_X_3_, X_1_^2^X_2_X_3_, and X_1_X_2_^2^X_3_ were shown to have significantly affected the pileus width (Additional file 1: Table S4). Interactions between X_2_X_3_ and X_1_X_2_^2^X_3_ may significantly reduce the pileus width. Interactions between X_1_X_2_, X_1_^2^X_2_X_3_ may significantly increase the pileus width.

### Effects of different agro-residue formulations on mycelial growth rate

With the statistical analysis of the mycelial growth rate for each substrate formula (Additional file 1: Table S5), the following regression model between the mycelial growth rate and each ingredient was developed:

$$\begin{aligned} Y & = 0.39X_{1} + 0.38X_{2} + 0.33X_{3} - 0.18X_{1} X_{2} - 0.16X_{1} X_{3} - 0.004568X_{2} X_{3} \\ & \quad + 0.18X_{1}^{2} X_{2} X_{3} - 9.22X_{1} X_{2}^{2} X_{3} + 7.34X_{1} X_{2} X_{3}^{2} \\ \end{aligned}$$

$$\left( R^{2} = 0.9906 \right)$$

The linear correlation coefficient for the pileus width was relatively high at R^2^ = 0.9906. As seen in the regression model, the equation coefficients were K (X_1_) = 0.39 > K (X_2_) = 0.38 > K (X_3_) = 0.33. This indicated that wheat straw had the greatest degree of contribution to the mycelial growth rate of *P. pulmonarius*.

The interactions between X_1_X_2_, X_1_X_3_, X_1_X_2_^2^X_3_, and X_1_X_2_X_3_^2^ were shown to have significantly affected the mycelial growth rate. It was concluded that the mycelial growth rate may be changed by the appropriate selection of the X_1_, X_2_, and X_3_ levels (Additional file 1: Table S5). Interactions between X_1_X_2_, X_1_X_3_, and X_1_X_2_^2^X_3_ may significantly reduce the mycelial growth rate, and X_1_X_2_X_3_^2^ may significantly increase the mycelial growth rate.

### Effects of different agro-residue substrate formulations on yield

With the statistical analysis of the yield for each substrate formula (Additional file 1: Table S6), the following regression model between the yield and each ingredient was developed:

$$\begin{aligned} Y & = 2037.37X_{1} + 2498.48X_{2} + 2916.14X_{3} + 1256.89X_{1} X_{2} - 1163.46X_{1} X_{3} - 74.92X_{2} X_{3} \\ & \quad + 80178.75X_{1}^{2} X_{2} X_{3} - 11917.38X_{1} X_{2}^{2} X_{3} - 20445.90X_{1} X_{2} X_{3}^{2} \\ \end{aligned}$$

$$\left( {R^{2} = 0.8747} \right)$$

Using the regression model, the predicted yield was calculated for each substrate formula, and the measured yield and the predicted yield were found to be very similar. As seen in the regression model, the equation coefficients were K (X_3_) = 2916.14 > K (X_2_) = 2498.48 > K (X_1_) = 2037.37, which indicated that the degree of contribution of each ingredient to the yield was as follows: X_3_ (soybean straw) > X_2_ (corn straw) > X_1_ (wheat straw). This result indicated that soybean straw had the greatest degree of contribution to the yield of *P. pulmonarius*.

The interactions between X_1_X_2_, X_1_X_3_, X_1_^2^X_2_X_3_, and X_1_X_2_X_3_^2^ were shown to have significantly affected the yield (Additional file 1: Table S6). Interactions between X_1_X_3_ and X_1_X_2_X_3_^2^ may significantly reduce the yield, and interactions between X_1_X_2_ and X_1_^2^X_2_X_3_ may significantly increase the yield.

### High-yielding formula and verification test

Using the yield, mycelial growth rate, stipe length, pileus length, pileus width, and the time to harvest as the evaluation indices, and setting the variation range and expected response value of each ingredient, a steepest slope prediction was carried out based on the regression equation beginning from a random combination, and a high yield comprehensive formula with the agro-residues was generated. The resulting substrate formula was as follows: 51.1% wheat straw (X_1_), 25.7% corn straw (X_2_), and 23.2% soybean straw. These values were then multiplied by the coefficient of 79.0% and thus converted to the final values of 40.4% wheat straw, 20.3% corn straw, 18.3% soybean straw, and 20.0% wheat bran and 1.0% light CaCO_3_ combined, which is the high yield comprehensive (HC). Using this substrate formula, the measured yield (first mushroom) reached 82.30 g/bag, which was similar to the predicted fitted value. These results showed that by using this formula, the yield (first mushroom) was increased by 21.8% compared to the CK (67.67 g/bag), and the BE was 15.2% greater than that of the CK (HC:118.6%; CK:103.4%). The mycelial growth rate, single-fruiting-body stipe length, stipe width, pileus length, pileus width, pileus thickness, time to bud formation, time to harvest, and the reproductive stage, as they pertain to the high yield comprehensive formula, were also examined. The single-fruiting-body pileus length (69 mm), pileus width (87 mm), and pileus thickness (23 mm) of *P. pulmonarius* for the high yield comprehensive formula were greater than those of the CK, which were 68 mm, 66 mm, and 20 mm, respectively. However, the time to bud formation of the high yield comprehensive formula was delayed by 4 days compared to the CK, even when the growth cycle was started 2 days in advance. Given this delay, the reproductive stage of the high yield comprehensive formula was 6 days shorter than that of the CK (Additional file 1: Table S7).

## Discussion

Several edible fungi have traditionally been cultivated on forest wood (sawdust); however, due to the recent efforts to manage forest resources, the use of agro-forestry products for its cultivation is no longer considered to be sustainable (Song et al. 2018). Wood rot fungus straw is being utilized for the production of *Pleurotus ostreatus*, *Pleurotus eryngii*, *Pleurotus cystidiosus*, *Lentinula edodes* and *Flammulina velutipes,* which can be cultivated with agro-residues, such as crop straws, either with composting or non-composting (Moonmoon et al. 2010; Hoa et al. 2015; Pedri et al. 2015; Rezaeian and Pourianfar. 2016; Papadaki et al. 2019). However, to date, there has been no study focused on optimizing the substrate formula for cultivating *Pleurotus* by the simple lattice method. *Pleurotus* primarily produces laccase and is one of the most widely cultivated, edible fungi in the world (Souza et al. 2002; Zhang et al. 2011).

The design of the simple lattice method is a basic mixing design method. Through regression analysis, the quantitative relationship between the ratio of the main material matrix and the evaluation index can be obtained, and therefore the optimum formula can be achieved (Yang et al. 2016). Consequently, in this study, the use of different agro-residues (wheat straw, rice straw, soybean straw, corn straw, corn cob, peanut straw, and rape straw) was investigated to find an substrate to replace sawdust and cottonseed hulls for cultivating *P. pulmonarius*. A statistical model for optimizing production and evaluating the effects of the alternative substrate formula on yield, mycelial growth rate, stipe length, pileus length, pileus width, and time to harvest was also described.

In the present study, three kinds of common agro-residues, wheat straw, corn straw, and soybean straw, were used as the main ingredients. The yield, mycelial growth rate, stipe length, pileus length, pileus width, and time to harvest were analyzed, and then optimized by the simple lattice method. In respect to the time to harvest, it was demonstrated that when the three substrates were used separately, the growth period associated with the soybean straw substrate was the shortest, followed by the corn straw, and the wheat straw. It was concluded that soybean straw may be beneficial in shortening the cultivation cycle of *P. pulmonarius*. Furthermore, the time to harvest was shortest when 100% soybean straw was used as the primary ingredient. The mycelial growth rate was the greatest on the wheat straw, followed by corn straw and soybean straw. The mushroom yield was the greatest when grown on the soybean straw, followed by corn straw and wheat straw (Additional file 1: Table S8). These results indicated that different substrate components had varying effects on several important agronomic traits. This may be attributed to their different nutritional components and structures, such as the differing contents of cellulose and lignin (Naraian et al. 2009). The carbon to nitrogen ratio (C/N) of the wheat straw, corn straw, and soybean straw was 70.92, 39.30, and 75.03, respectively (Miu 2015; Wu et al. 2018). The cellulose content of the wheat straw, corn straw, and soybean straw was 39.26%, 37.15%, and 30.83%, respectively, and the lignin content of the wheat straw, corn straw, and soybean straw was 22.10%, 22.14%, and 32.80%, respectively (Miu 2015; Wu et al. 2018). The cellulose content of the soybean straw was the lowest, but its lignin content was the highest. It can be inferred that the substrate with higher lignin content could be more conducive to growth in terms of stipe length, pileus length, pileus width, yield increases, and by decreasing the time to harvest of *P. pulmonarius*. The difference in the C/N ratio is also an important factor affecting the evaluation index of edible fungi (Yang et al. 2013). The soybean straw had the greatest influence the time to harvest, stipe length, pileus length, pileus width, and yield, indicating that the soybean straw is a suitable cultivation matrix. However, in addition to the soybean straw, wheat straw and corn straw can also be used together to maximize this impact.

In conclusion, this study demonstrated that the overall yield of *P. pulmonarius* could be improved by optimizing the substrate formula for cultivation; thus, we propose using alternatives to sawdust and cottonseed hulls. The amount of the agro-residue (wheat straw, soybean straw, and corn straw) used had a significant influence on the dependent variables. Wheat straw had the greatest effect on mycelial growth rate, followed by corn straw and soybean straw. Soybean straw had the greatest effect on time to harvest, stipe length, pileus length, pileus width, and yield, followed by corn straw and wheat straw. By verifying the optimized high yield comprehensive substrate formula, it was concluded that these agro-residues (i.e., wheat straw, corn straw, and soybean straw) may be used to replace sawdust and cottonseed hulls as the main cultivation substrates of *P. pulmonarius*. The findings of the present study provide a theoretical basis for the “replacing wood for grass” project on edible mushroom cultivation. Future research should explore the mechanisms by which wood rot fungi decompose grasses, and screen to select candidate wood rot fungi strains that can be cultivated with agricultural residues.

## Supplementary information

**Additional file 1: Table S1.** Variance Analysis of the Quadratic Polynomial Regression Model for Time to Harvest. **Table S2.** Variance Analysis of the Quadratic Polynomial Regression Model for Stipe Length. **Table S3.** Variance Analysis of the Quadratic Polynomial Regression Model for Pileus Length. **Table S4.** Variance Analysis of the Quadratic Polynomial Regression Model for Pileus Width. **Table S5.** Variance Analysis of the Quadratic Polynomial Regression Model for Mycelial Growth Rate. **Table S6.** Variance Analysis of the Quadratic Polynomial Regression Model for Yield. **Table S7.** Comparison of Main Agronomic Traits Between the HC Formula and the CK Substrate Formula. **Table S8.** The Influence of the Three Agro-residues as 100% of the Main Ingredient on Each Evaluation Index.

## Data Availability

The datasets supporting the conclusions of this article are included within the article.

## Abbreviations

BE: biological efficiency
CK: control
OFAT: one-factor-at-a-time
PDA: potato dextrose agar
SNK: Student–Newman–Keuls
