# Supplementary material for: Optimization of agro-residues as substrates for Pleurotus pulmonarius production
Source: AMB Express. 2019 Nov 14;9:184. doi: 10.1186/s13568-019-0907-1 (PMC6856248; doi:10.1186/s13568-019-0907-1)
Supplement: Supplementary file 1 — Additional file 1: Table S1. Variance Analysis of the Quadratic Polynomial Regression Model for Time to Harvest. Table S2. Variance Analysis of the Quadratic Polynomial Regression Model for Stipe Length. Table S3. Variance Analysis of the Quadratic Polynomial Regression Model for Pileus Length. Table S4. Variance Analysis of the Quadratic Polynomial Regression Model for Pileus Width. Table S5. Variance Analysis of the Quadratic Polynomial Regression Model for Mycelial Growth Rate. Table S6. Variance Analysis of the Quadratic Polynomial Regression Model for Yield. Table S7. Comparison of Main Agronomic Traits Between the HC Formula and the CK Substrate Formula. Table S8. The Influence of the Three Agro-residues as 100% of the Main Ingredient on Each Evaluation Index. [file 13568_2019_907_MOESM1_ESM.pptx]

## Slide 1
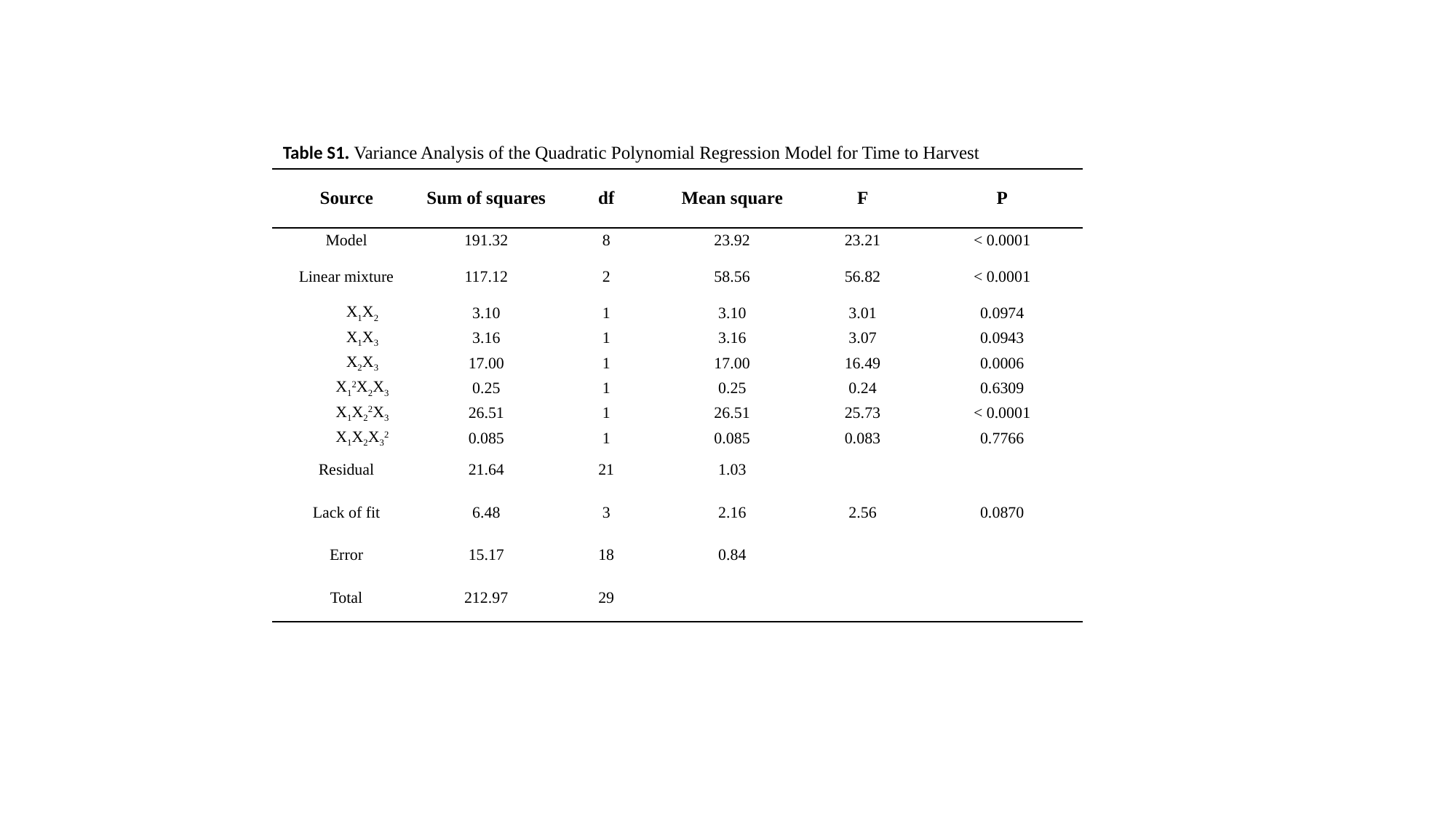

Table S1. Variance Analysis of the Quadratic Polynomial Regression Model for Time to Harvest
| Source | Sum of squares | df | Mean square | F | P |
| --- | --- | --- | --- | --- | --- |
| Model | 191.32 | 8 | 23.92 | 23.21 | < 0.0001 |
| Linear mixture | 117.12 | 2 | 58.56 | 56.82 | < 0.0001 |
| X1X2 | 3.10 | 1 | 3.10 | 3.01 | 0.0974 |
| X1X3 | 3.16 | 1 | 3.16 | 3.07 | 0.0943 |
| X2X3 | 17.00 | 1 | 17.00 | 16.49 | 0.0006 |
| X12X2X3 | 0.25 | 1 | 0.25 | 0.24 | 0.6309 |
| X1X22X3 | 26.51 | 1 | 26.51 | 25.73 | < 0.0001 |
| X1X2X32 | 0.085 | 1 | 0.085 | 0.083 | 0.7766 |
| Residual | 21.64 | 21 | 1.03 | | |
| Lack of fit | 6.48 | 3 | 2.16 | 2.56 | 0.0870 |
| Error | 15.17 | 18 | 0.84 | | |
| Total | 212.97 | 29 | | | |

## Slide 2
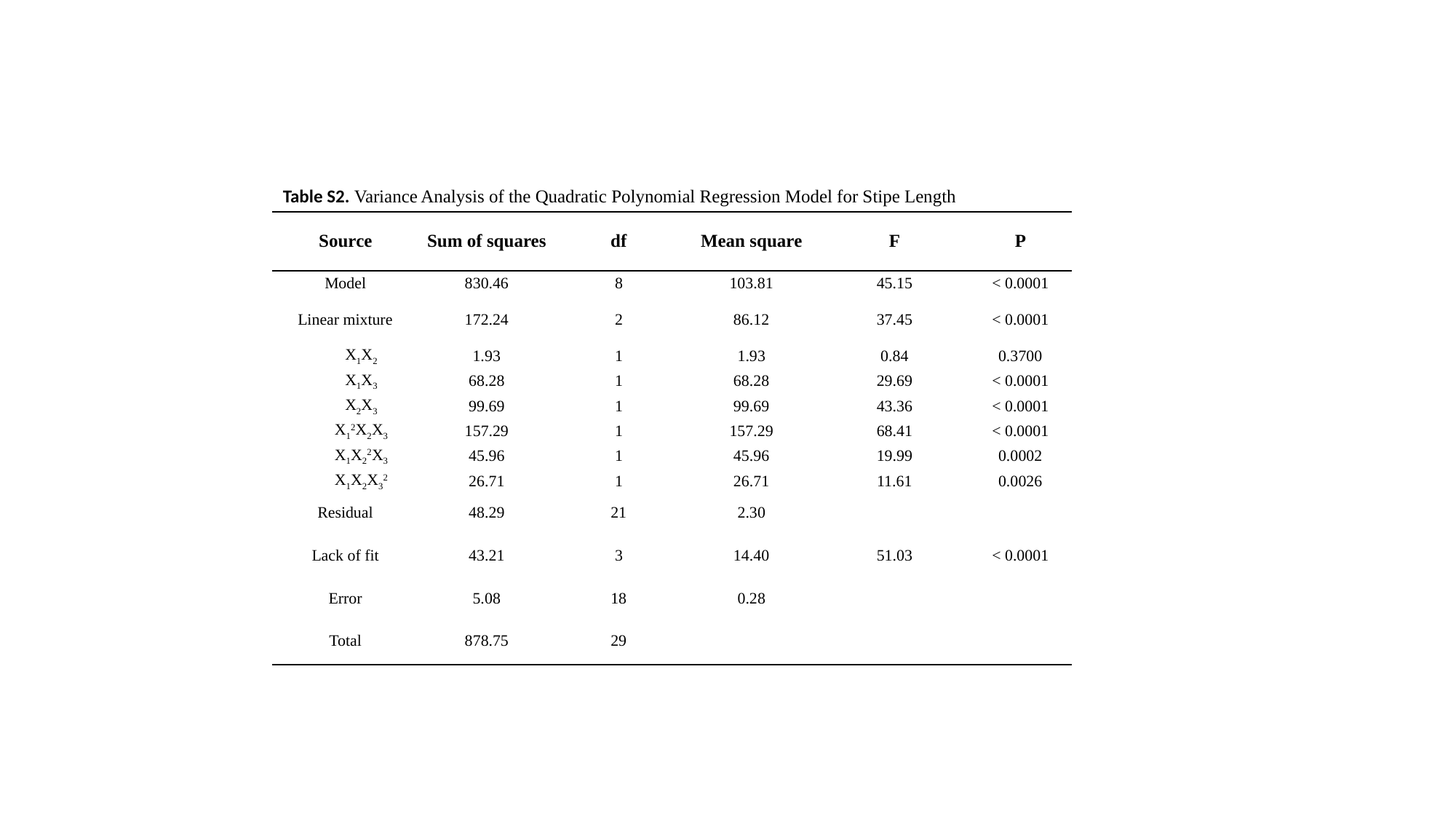

Table S2. Variance Analysis of the Quadratic Polynomial Regression Model for Stipe Length
| Source | Sum of squares | df | Mean square | F | P |
| --- | --- | --- | --- | --- | --- |
| Model | 830.46 | 8 | 103.81 | 45.15 | < 0.0001 |
| Linear mixture | 172.24 | 2 | 86.12 | 37.45 | < 0.0001 |
| X1X2 | 1.93 | 1 | 1.93 | 0.84 | 0.3700 |
| X1X3 | 68.28 | 1 | 68.28 | 29.69 | < 0.0001 |
| X2X3 | 99.69 | 1 | 99.69 | 43.36 | < 0.0001 |
| X12X2X3 | 157.29 | 1 | 157.29 | 68.41 | < 0.0001 |
| X1X22X3 | 45.96 | 1 | 45.96 | 19.99 | 0.0002 |
| X1X2X32 | 26.71 | 1 | 26.71 | 11.61 | 0.0026 |
| Residual | 48.29 | 21 | 2.30 | | |
| Lack of fit | 43.21 | 3 | 14.40 | 51.03 | < 0.0001 |
| Error | 5.08 | 18 | 0.28 | | |
| Total | 878.75 | 29 | | | |

## Slide 3
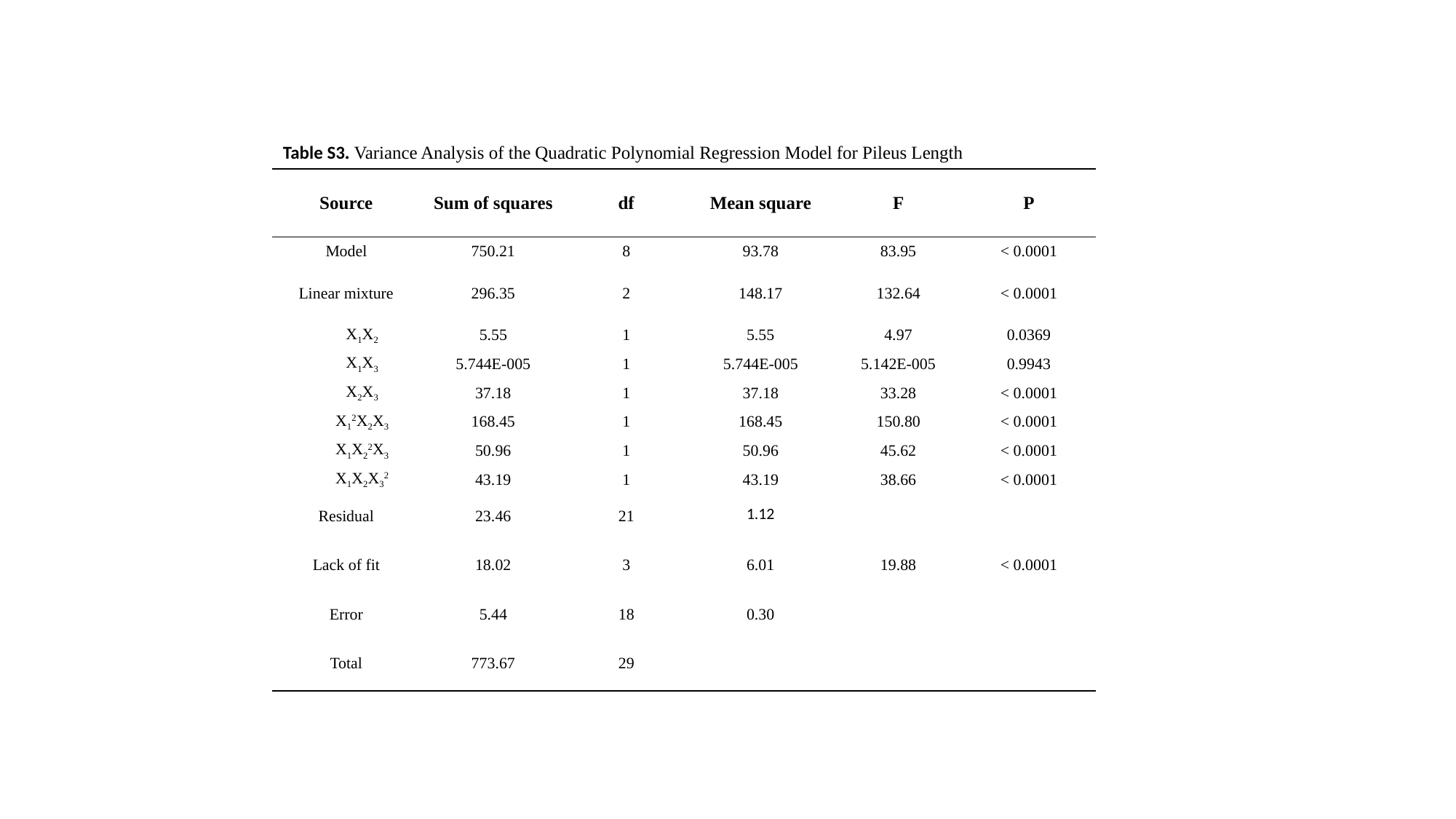

Table S3. Variance Analysis of the Quadratic Polynomial Regression Model for Pileus Length
| Source | Sum of squares | df | Mean square | F | P |
| --- | --- | --- | --- | --- | --- |
| Model | 750.21 | 8 | 93.78 | 83.95 | < 0.0001 |
| Linear mixture | 296.35 | 2 | 148.17 | 132.64 | < 0.0001 |
| X1X2 | 5.55 | 1 | 5.55 | 4.97 | 0.0369 |
| X1X3 | 5.744E-005 | 1 | 5.744E-005 | 5.142E-005 | 0.9943 |
| X2X3 | 37.18 | 1 | 37.18 | 33.28 | < 0.0001 |
| X12X2X3 | 168.45 | 1 | 168.45 | 150.80 | < 0.0001 |
| X1X22X3 | 50.96 | 1 | 50.96 | 45.62 | < 0.0001 |
| X1X2X32 | 43.19 | 1 | 43.19 | 38.66 | < 0.0001 |
| Residual | 23.46 | 21 | 1.12 | | |
| Lack of fit | 18.02 | 3 | 6.01 | 19.88 | < 0.0001 |
| Error | 5.44 | 18 | 0.30 | | |
| Total | 773.67 | 29 | | | |

## Slide 4
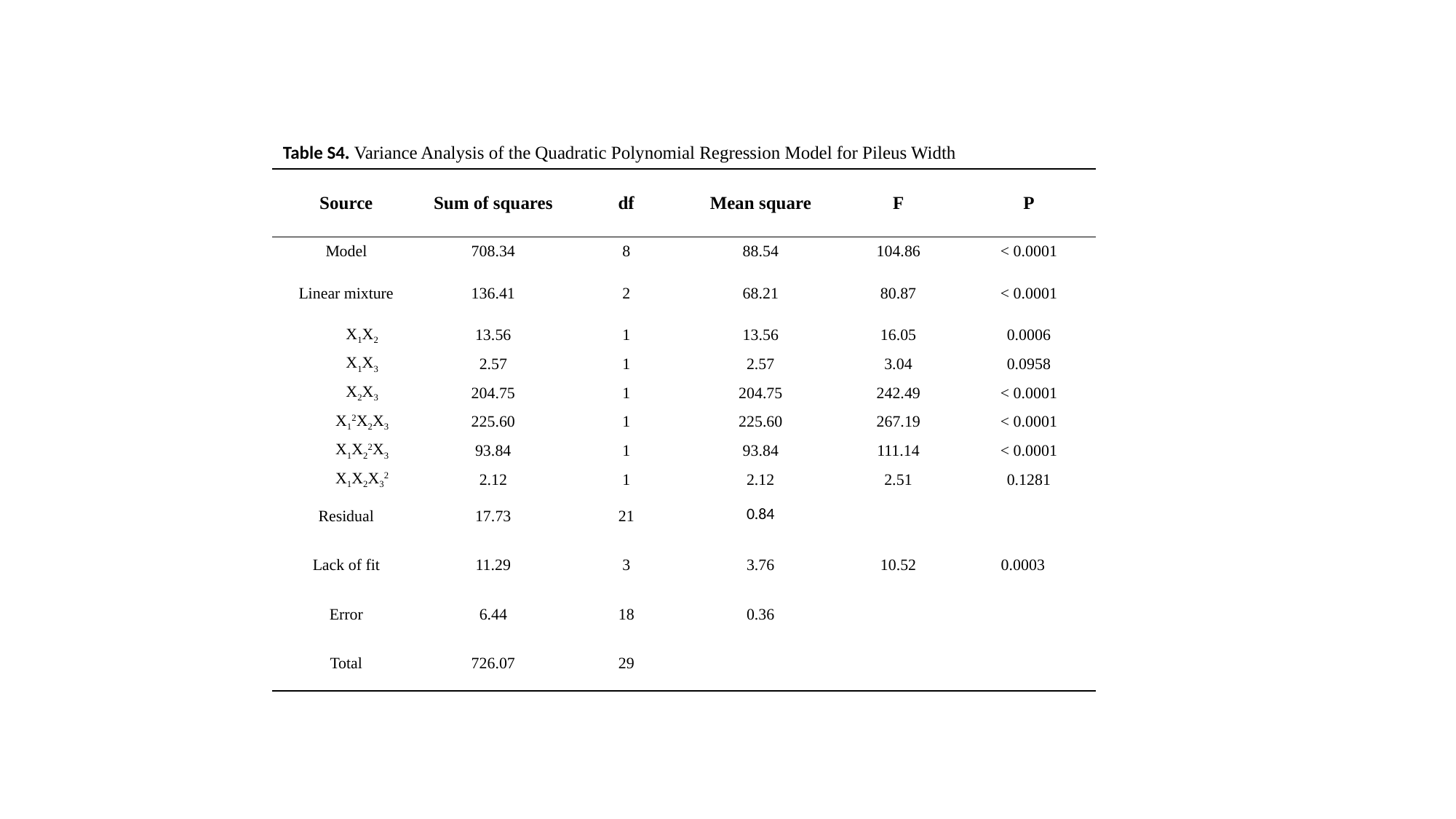

Table S4. Variance Analysis of the Quadratic Polynomial Regression Model for Pileus Width
| Source | Sum of squares | df | Mean square | F | P |
| --- | --- | --- | --- | --- | --- |
| Model | 708.34 | 8 | 88.54 | 104.86 | < 0.0001 |
| Linear mixture | 136.41 | 2 | 68.21 | 80.87 | < 0.0001 |
| X1X2 | 13.56 | 1 | 13.56 | 16.05 | 0.0006 |
| X1X3 | 2.57 | 1 | 2.57 | 3.04 | 0.0958 |
| X2X3 | 204.75 | 1 | 204.75 | 242.49 | < 0.0001 |
| X12X2X3 | 225.60 | 1 | 225.60 | 267.19 | < 0.0001 |
| X1X22X3 | 93.84 | 1 | 93.84 | 111.14 | < 0.0001 |
| X1X2X32 | 2.12 | 1 | 2.12 | 2.51 | 0.1281 |
| Residual | 17.73 | 21 | 0.84 | | |
| Lack of fit | 11.29 | 3 | 3.76 | 10.52 | 0.0003 |
| Error | 6.44 | 18 | 0.36 | | |
| Total | 726.07 | 29 | | | |

## Slide 5
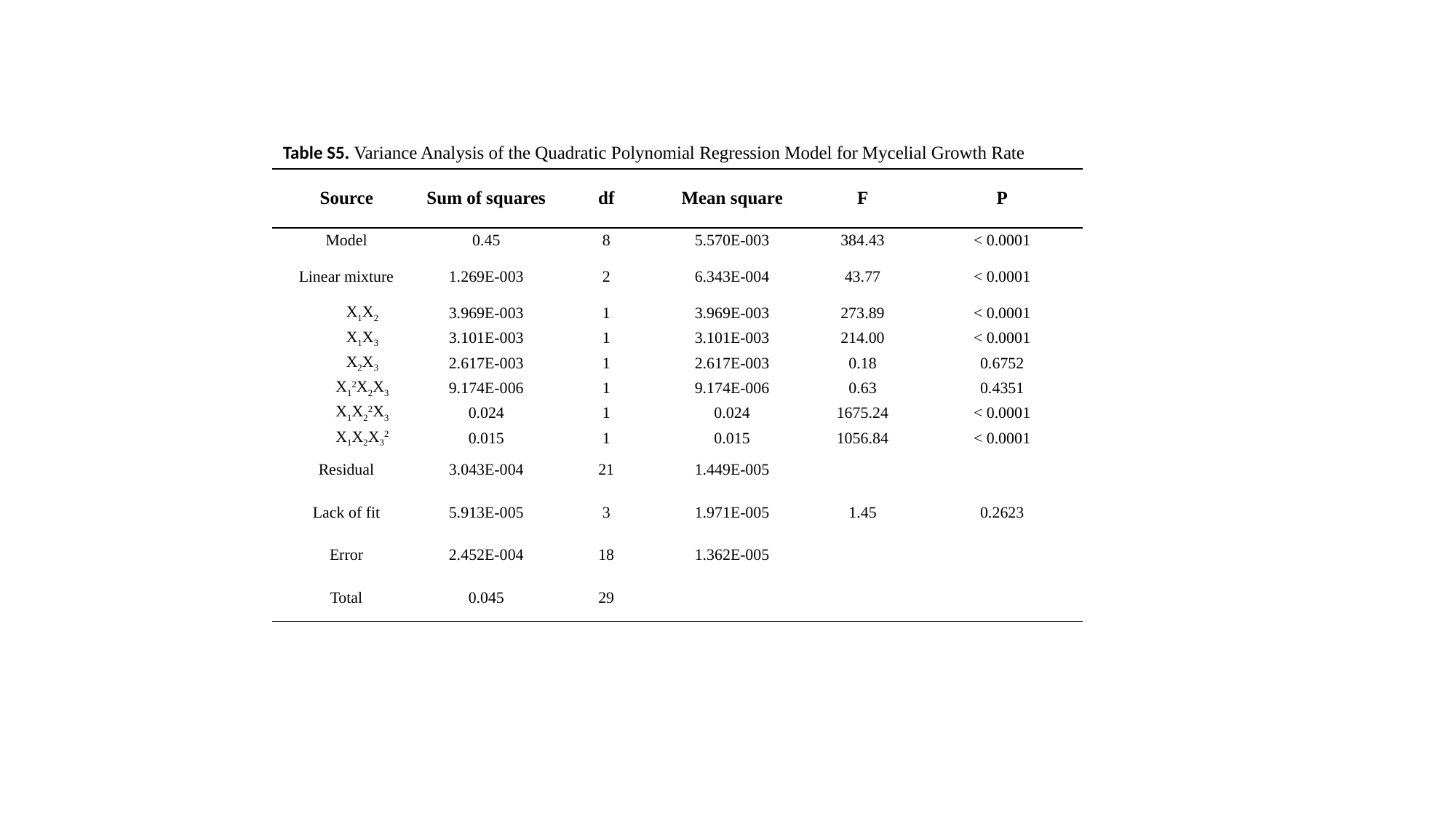

Table S5. Variance Analysis of the Quadratic Polynomial Regression Model for Mycelial Growth Rate
| Source | Sum of squares | df | Mean square | F | P |
| --- | --- | --- | --- | --- | --- |
| Model | 0.45 | 8 | 5.570E-003 | 384.43 | < 0.0001 |
| Linear mixture | 1.269E-003 | 2 | 6.343E-004 | 43.77 | < 0.0001 |
| X1X2 | 3.969E-003 | 1 | 3.969E-003 | 273.89 | < 0.0001 |
| X1X3 | 3.101E-003 | 1 | 3.101E-003 | 214.00 | < 0.0001 |
| X2X3 | 2.617E-003 | 1 | 2.617E-003 | 0.18 | 0.6752 |
| X12X2X3 | 9.174E-006 | 1 | 9.174E-006 | 0.63 | 0.4351 |
| X1X22X3 | 0.024 | 1 | 0.024 | 1675.24 | < 0.0001 |
| X1X2X32 | 0.015 | 1 | 0.015 | 1056.84 | < 0.0001 |
| Residual | 3.043E-004 | 21 | 1.449E-005 | | |
| Lack of fit | 5.913E-005 | 3 | 1.971E-005 | 1.45 | 0.2623 |
| Error | 2.452E-004 | 18 | 1.362E-005 | | |
| Total | 0.045 | 29 | | | |

## Slide 6
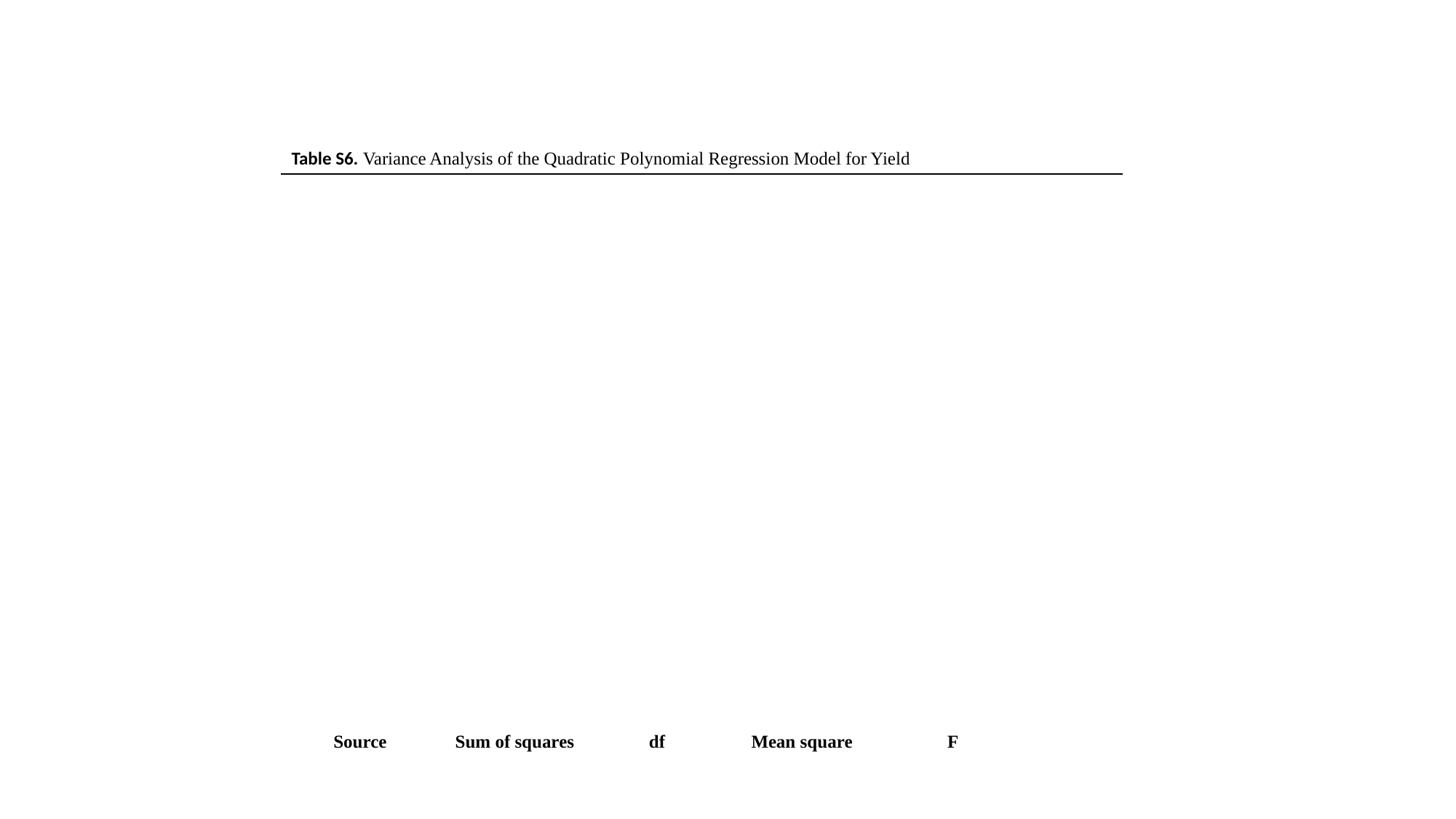

Table S6. Variance Analysis of the Quadratic Polynomial Regression Model for Yield
| Source | Sum of squares | df | Mean square | F | P |
| --- | --- | --- | --- | --- | --- |
| Model | 3.413E+006 | 8 | 4.267E+005 | 26.32 | <0.0001 |
| Linear mixture | 4.198E+005 | 2 | 2.099E+005 | 12.95 | 0.0002 |
| X1X2 | 1.981E+005 | 1 | 1.981E+005 | 12.22 | 0.0022 |
| X1X3 | 1.698E+005 | 1 | 1.698E+005 | 10.47 | 0.0040 |
| X2X3 | 703.91 | 1 | 703.91 | 0.043 | 0.8370 |
| X12X2X3 | 1.831E+006 | 1 | 1.831E+006 | 112.95 | <0.0001 |
| X1X22X3 | 40544.36 | 1 | 40544.36 | 2.50 | 0.1287 |
| X1X2X32 | 1.189E+005 | 1 | 1.189E+005 | 7.33 | 0.0132 |
| Residual | 3.405E+005 | 21 | 16213.62 | | |
| Lack of fit | 3.400E+005 | 3 | 1.133E+005 | 3840.09 | <0.0001 |
| Error | 531.17 | 18 | 29.51 | | |
| Total | 3.754E+006 | 29 | | | |

## Slide 7
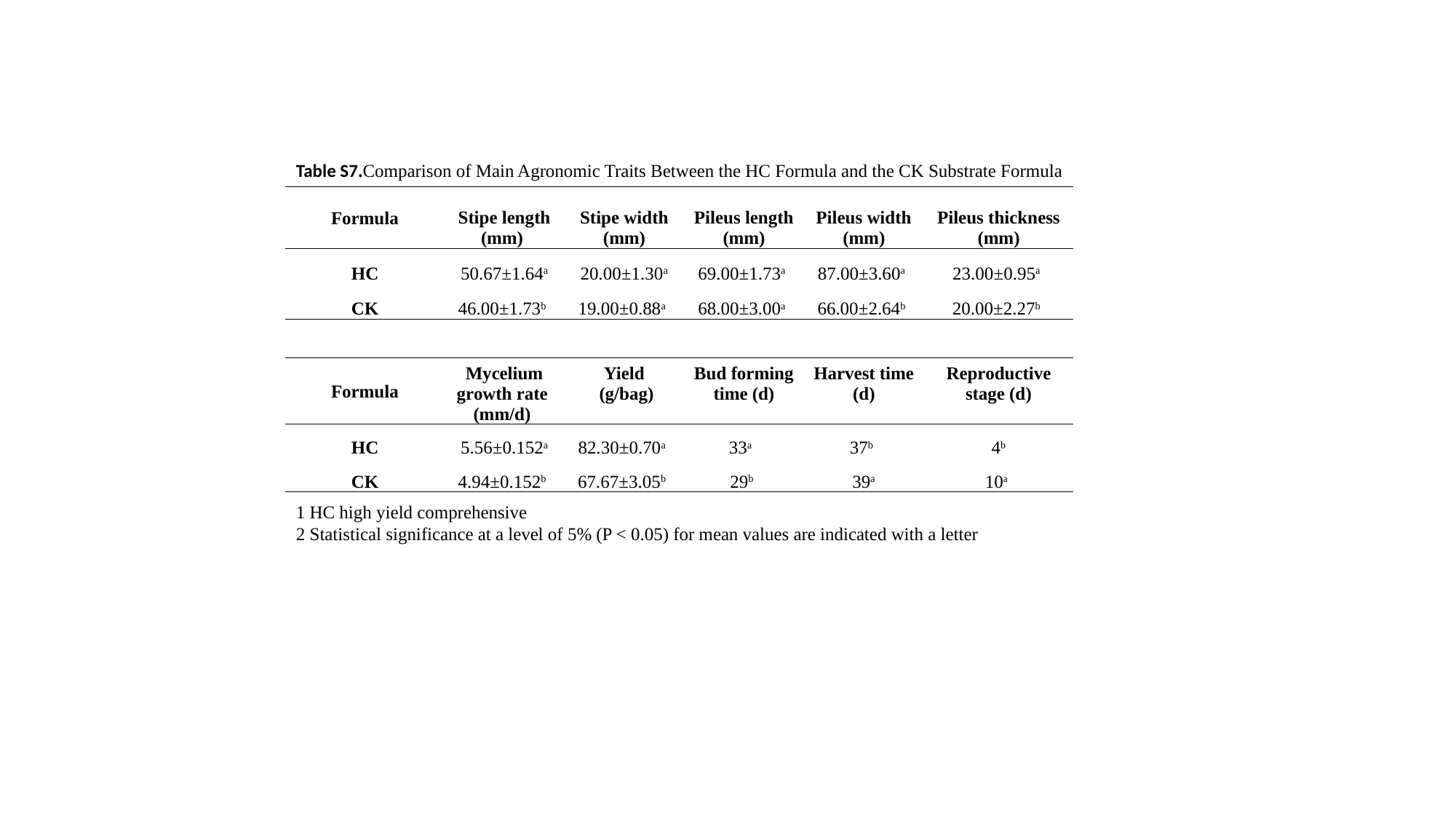

Table S7.Comparison of Main Agronomic Traits Between the HC Formula and the CK Substrate Formula
| Formula | Stipe length (mm) | Stipe width (mm) | Pileus length (mm) | Pileus width (mm) | Pileus thickness (mm) |
| --- | --- | --- | --- | --- | --- |
| HC | 50.67±1.64a | 20.00±1.30a | 69.00±1.73a | 87.00±3.60a | 23.00±0.95a |
| CK | 46.00±1.73b | 19.00±0.88a | 68.00±3.00a | 66.00±2.64b | 20.00±2.27b |
| Formula | Mycelium growth rate (mm/d) | Yield (g/bag) | Bud forming time (d) | Harvest time (d) | Reproductive stage (d) |
| --- | --- | --- | --- | --- | --- |
| HC | 5.56±0.152a | 82.30±0.70a | 33a | 37b | 4b |
| CK | 4.94±0.152b | 67.67±3.05b | 29b | 39a | 10a |
1 HC high yield comprehensive
2 Statistical significance at a level of 5% (P < 0.05) for mean values are indicated with a letter

## Slide 8
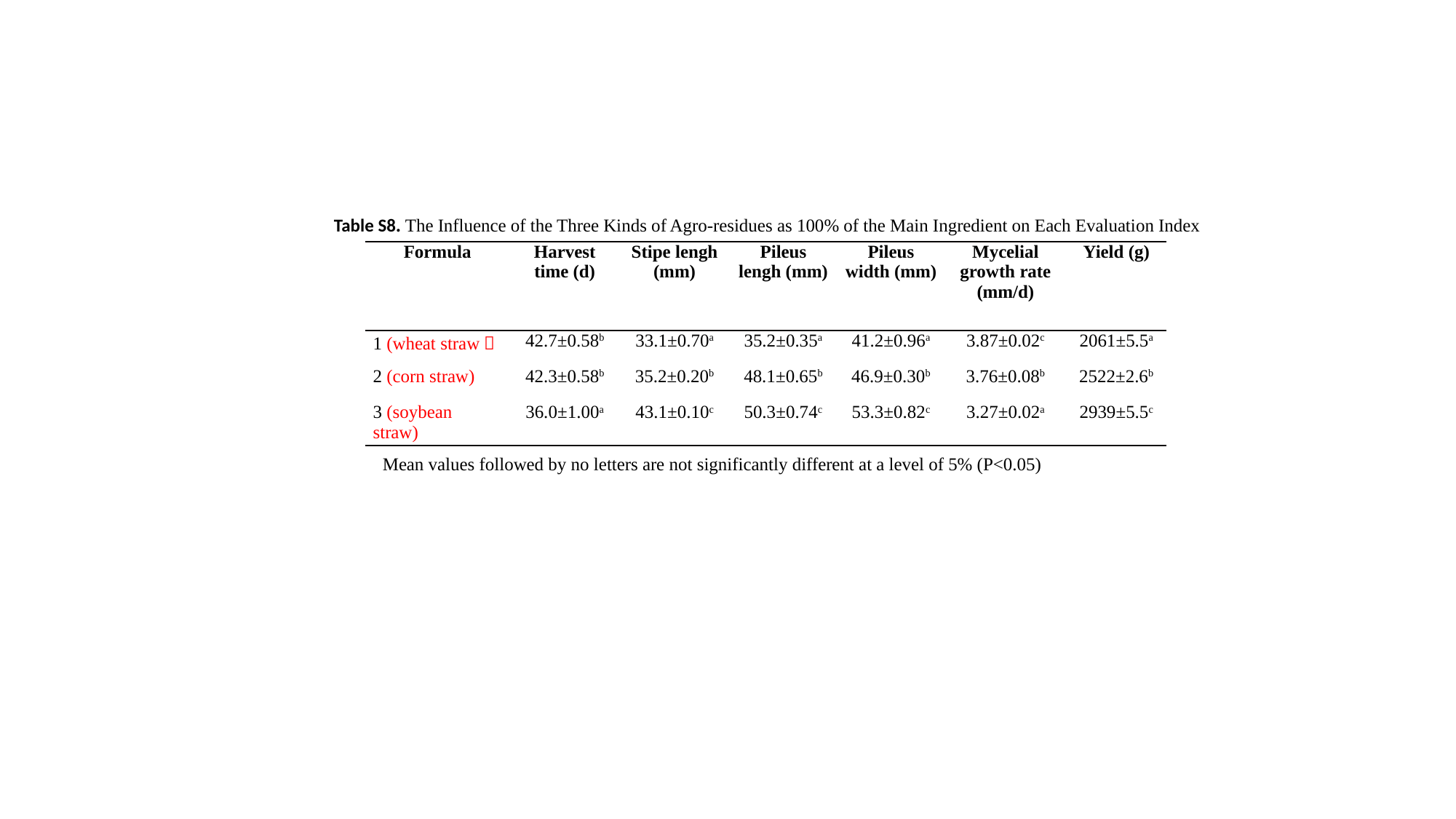

Table S8. The Influence of the Three Kinds of Agro-residues as 100% of the Main Ingredient on Each Evaluation Index
| Formula | Harvest time (d) | Stipe lengh (mm) | Pileus lengh (mm) | Pileus width (mm) | Mycelial growth rate (mm/d) | Yield (g) |
| --- | --- | --- | --- | --- | --- | --- |
| 1 (wheat straw） | 42.7±0.58b | 33.1±0.70a | 35.2±0.35a | 41.2±0.96a | 3.87±0.02c | 2061±5.5a |
| 2 (corn straw) | 42.3±0.58b | 35.2±0.20b | 48.1±0.65b | 46.9±0.30b | 3.76±0.08b | 2522±2.6b |
| 3 (soybean straw) | 36.0±1.00a | 43.1±0.10c | 50.3±0.74c | 53.3±0.82c | 3.27±0.02a | 2939±5.5c |
 Mean values followed by no letters are not significantly different at a level of 5% (P<0.05)
